# Nuclear entrapment and extracellular depletion of PCOLCE is associated with muscle degeneration in oculopharyngeal muscular dystrophy

**DOI:** 10.1186/1471-2377-13-70

**Published:** 2013-07-01

**Authors:** Vered Raz, Ellen Sterrenburg, Samantha Routledge, Andrea Venema, Barbara M van der Sluijs, Capucine Trollet, George Dickson, Baziel GM van Engelen, Silvère M van der Maarel, Michael N Antoniou

**Affiliations:** 1 Center for Human and Clinical Genetics, Leiden University Medical Center, Einthovenweg 20, Leiden, The Netherlands; 2 Gene Expression and Therapy Group, King’s College London School of Medicine, Department of Medical and Molecular Genetics, 8th Floor Tower Wing, Guy’s Hospital, London SE1 9RT, UK; 3 Neuromuscular Centre Nijmegen, Institute of Neurology, Radboud University Nijmegen Medical Centre, P.O. Box 9101, 6500, Nijmegen, HB, The Netherlands; 4 School of Biological Sciences, Royal Holloway, University of London, Egham, Surrey TW20 0EX, UK; 5 Institut de Myologie, INSERM U974 and CNRS UMR 7215, Paris, France

**Keywords:** OPMD, Extracellular matrix, PCOLCE, Muscle fibrosis, PABPN1

## Abstract

**Background:**

Muscle fibrosis characterizes degenerated muscles in muscular dystrophies and in late onset myopathies. Fibrotic muscles often exhibit thickening of the extracellular matrix (ECM). The molecular regulation of this process is not fully understood. In oculopharyngeal muscular dystrophy (OPMD), an expansion of an alanine tract at the N-terminus of poly(A)-binding protein nuclear 1 (PABPN1) causes muscle symptoms. OPMD patient muscle degeneration initiates after midlife, while at an earlier age carriers of alanine expansion mutant PABPN1 (expPABPN1) are clinically pre-symptomatic. OPMD is characterized by fibrosis in skeletal muscles but the causative molecular mechanisms are not fully understood.

**Methods:**

We studied the molecular processes that are involved in OPMD pathology using cross-species mRNA expression profiles in muscles from patients and model systems. We identified significant dysregulation of the ECM functional group, among which the procollagen C-endopeptidase enhancer 1 gene (*PCOLCE*) was consistently down-regulated across species. We investigated PCOLCE subcellular localization in OPMD muscle samples and OPMD model systems to investigate any functional relevance of PCOLCE down-regulation in this disease.

**Results:**

We found that muscle degeneration in OPMD is associated with PCOLCE down-regulation. In addition to its known presence at the ECM, we also found PCOLCE within the nucleus of muscle cells. PCOLCE sub-cellular localization changes during myoblast cell fusion and is disrupted in cells expressing mutant expPABPN1. Our results show that PCOLCE binds to soluble PABPN1 and co-localizes with aggregated PABPN1 with a preference for the mutant protein. In muscle biopsies from OPMD patients we find that extracellular PCOLCE is depleted with its concomitant enrichment within the nuclear compartment.

**Conclusions:**

PCOLCE regulates collagen processing at the ECM. Depletion of extracellular PCOLCE is associated with the expression of expPABPN1 in OPMD patient muscles. PCOLCE is also localized within the nucleus where it binds to PABPN1, suggesting that PCOLCE shuttles between the ECM and the nucleus. PCOLCE preferentially binds to expPABPN1. Nuclear-localized PCOLCE is enriched in muscle cells expressing expPABPN1. We suggest that nuclear entrapment of PCOLCE and its extracellular depletion represents a novel molecular mechanism in late-onset muscle fibrosis.

## Background

The extracellular matrix (ECM) plays a key role in regulating homeostasis and tissue repair. Wound healing and fibrosis are highly regulated by the ECM [1]. The ECM surrounding skeletal muscle tissue maintains cellular function and fibre structure, and further provides an environment for muscle fibre contraction. Defects or deficiencies in molecules that reside in the ECM can cause skeletal muscle myopathy and muscle fibrosis [2]. Fibrotic muscle tissue exhibits an excess accumulation of collagen and other ECM components [3], leading to aberrant muscle fibre function [4]. The role of the ECM in muscle function has been extensively studied in Duchene muscular dystrophy (DMD). DMD is caused by mutations in the gene that encodes Dystrophin, a mechanical transducer muscle protein that transmits external stimuli into the cell. When Dystrophin is mutated, signals from the ECM are poorly transduced into the cell [5]. Nevertheless, the molecular mechanisms that are associated with changes in the ECM in different myopathies are not fully understood.

Oculopharyngeal muscular dystrophy (OPMD) is a late-onset, usually autosomal dominantly inherited myopathy [6]. In OPMD muscle weakness is progressive and initially affects muscles of the eyelid, throat and proximal limbs. OPMD patients harbour a triplet repeat expansion mutation in the gene encoding poly(A)-binding protein nuclear 1 (*PABPN1*), leading to an expansion of the poly-alanine (poly-Ala) tract at the *N*-terminus of PABPN1 [7]. Alanine expanded PABPN1 (expPABPN1) is prone to aggregation and forms insoluble inclusions within the nuclei of skeletal muscle fibres in OPMD patients [8]. In addition, OPMD muscles contain rimmed vacuoles, which are a characteristic of fibrotic muscles [9]. Fibrotic muscles were also reported in a mouse model of OPMD that over expresses expPABPN1 in skeletal muscles [10,11]. To date, ECM defects and their potential contribution to OPMD muscle symptoms have not been investigated.

Here we report deregulation of the ECM functional group in OPMD *Vastus lateralis (VL)* muscles. Dysrgulation of the ECM was consistent in model systems for OPMD. We focused on procollagen C-proteinase enhancer 1 (*PCOLCE*; PCPE-1) as it was found to be consistently deregulated in OPMD patients and in a muscle cell model system of this condition. PCOLCE is predominantly localised at the ECM, where it functions as a positive regulator of collagen deposition [12]. Nuclear-localised PCOLCE co-immunoprecipitates with soluble expPABPN1 and co-localises with aggregated PABPN1. In both cell and animal model systems of OPMD that express expPABPN1, PCOLCE retention in the nucleus was concomitant with reduced accumulation in the ECM. We suggest that nuclear entrapment of PCOLCE is associated with its extracellular depletion, and hence represents a novel mechanism by which this protein is involved in muscle fibrosis.

## Methods

### Cell culture and collection of muscle biopsies

An ImmortoMouse-derived myoblast cell clone, designated IM2, was produced as previously described [13], and used to generate stably transfected clones expressing either normal Ala10-*humanPABPN1*-FLAG (Ala10) or mutant Ala17-*humanPABPN1*-FLAG (Ala17) [14]. Cells were maintained in a proliferating medium containing DMEM (Invitrogen, Carlsbad, CA, USA), supplemented with 20% foetal calf serum (FCS) (Invitrogen), 0.5% chicken embryo extract (PAA laboratories, Somerset, UK), 100 U/ml penicillin/streptomycin, 2 mM L-glutamine and 20 U/ml IFNγ (HyCult Biotech, Uden, The Netherlands) at 33°C in a humidified 10% CO_2_-air atmosphere. Myoblast fusion was conducted with confluent cultures maintained in fusion medium consisting of DMEM containing 5% horse serum without IFNγ and incubation at 37°C in a humidified 5% CO_2_-air atmosphere for up to 5 days. Details of the generation of the Ala10 and Ala17 IM2-derived clones used in this study have been previously described [14].

All human *VL* muscle biopsies analysed in this study were collected at Radboud Hospital, Nijmegen, Netherlands and are listed in Table 1. *VL* muscle biopsies were collected using the Bergstrom needle procedure after approval from the medical ethical committee at Arnhem Nijmegen (CMO nr.:2005/189). Biopsy sampling from mice was conducted at Royal Holloway College, University of London, Egham, UK, following ethical approval from the Ethical Review Board at Royal Holloway (University of London) and the UK government Home Office, under license to Prof G Dickson (project nr. PPL70/7008: Personal license reference number – PIL 70/7682).

**Table 1:** Clinical description of OPMD patients and pre-symptomatic carriers contributing to this study

**Symptomatic**			**Pre-symptomatic**		
**Age**	**MRC score**	**Gender**	**Age**	**MRC score**	**Gender**
49	4	Female	37	ND	Female
54	5	Female	37	ND	Female
57	4	Female	38	ND	Male
59	5	Male	39	ND	Female
60	4	Female	39	ND	Female
60	4.5	Female	41	ND	Female
66	4.5	Male			
68	3.5	Male			
69	4.5	Female			

The age of medical examination and biopsy sampling is indicated. Medical research conceal (MRC) score is a non-linear clinical measure for muscle strength. MRC was determined in left and right quadriceps and the average of both legs is shown. An MRC smaller than 5 indicates clinical muscle weakness. Clinical investigation was carried out by a single doctor and was performed at the same day of biopsy sampling. Clinical muscle weakness is not found in pre-symptomatic and in controls subjects (ND).

### Protein analysis and detection procedures

*Total protein extracts* from unfused myoblast and fused myotube cultures were produced by direct lysis in sodium dodecyl sulphate (SDS) loading buffer (50 mM Tris pH 6.8, 10 mM DTT, 2% SDS, 0.1% bromophenol blue, 20% glycerol). Samples were resolved by 10% polyacrylamide gel electrophoresis (PAGE) under denaturing conditions followed by Western blot analysis.

*Immonoprecipitation* of soluble proteins was conducted with protein extracts from 4-day fused myoblast cultures. Proteins were extracted with a buffer containing 150 mM NaCl, 50 mM Tris pH7.5, 0.1% Tween and protease inhibitor cocktail (Sigma-Aldrich, Saint Louis, Missouri, USA). FLAG-tagged PABPN1 protein in these extracts was bound with rabbit-anti FLAG (Sigma-Aldrich) antibodies and precipitated with Protein-A Sepharose (Sigma-Aldrich). After extensive washing with decreasing NaCl concentrations (150-0 mM), loading buffer was added and samples resolved by 10% SDS-PAGE followed by Western blot analysis.

*Western blot analysis* was conducted by a transfer of SDS-PAGE resolved products onto PVDF membranes. Detection of primary antibodies was conducted after incubation with horseradish peroxidase (HRP)-conjugated secondary antibodies with the Amersham Hybond ECL chemiluminescence detection system (GE Healthcare Bio-Sciences AB, Uppsala, Sweden).

*Histological staining and immunofluorescence analysis* of myotube cultures was performed as previously described [14]. Human skeletal muscle cryosections were generated from biopsy samples of *VL* muscles from both healthy controls and OPMD patients after informed consent. Mouse muscle sections were generated from *Tibialis anterior* (*TA*) muscles from wild type or A17.1 OPMD model mice [10] at 12, 18 and 26 weeks of age. Sections were fixed in PBS/3.7% formaldehyde for 30 minutes followed by permeabilisation with 1% Triton-X100 for 30 min and washing with PBS following antibody incubations as above.

Treatment with 1 M KCl was for 20 minutes and preceded tissue fixation. Human muscle sections were incubated with 0.3% Sudan Black B (Sigma-Aldrich) for 10 min to eliminate background from intrinsic autofluorescent pigments. After completion of antibody staining steps, samples were overlaid with cover-slips mounted in Vectashield (Vector, Peterborough, UK) or Vectashield containing 4′-6-diamidino-2-phenylindole (Sigma-Aldrich) at a concentration of 3 ng/l. Images were recorded with a Zeiss Axiovert (model 135TV) fluorescence microscope equipped with a 100-W mercury arc lamp and a 40x/1.3 NA plan Apo objective. Single confocal sections were obtained with a Leica TCS/SP2 confocal microscope system equipped with a 100x/1.4 NA plan Apo objective.

For histological staining, human specimens were processed for light microscopic investigation and stained with haematoxylin-eosin.

Primary antibodies used were: mouse monoclonal anti-FLAG M2 (Sigma-Aldrich); mouse monoclonal anti-muscle actin (MSA) (Novocastra, Newcastle upon Tyne, UK); mouse monoclonal anti-α-Tubulin (Sigma-Aldrich); mouse anti-ubiquitin, FK2, (AbCam, Cambridge, UK); rabbit polyclonal anti-Pcolce (AbCam and Sigma-Aldrich); mouse anti-Dysferlin (Novocastra); anti-MBNL1 (donated by Dr. T.A. Cooper Baylor College of Medicine, Houston, USA), Myc-tagged 3 F5 llama single chain antibody [15], which is recognised with mouse-anti-Myc clone SE50 (Sigma-Aldrich) at dilutions 1:1000–3000. Alexa 488-, Alexa 430- or Alexa 594- conjugated secondary antibodies against primary antibodies were obtained from Molecular Probes (Invitrogen).

Image quantification was performed with ImageJ application (http://rsbweb.nih.gov/ij/).

### RNA expression and transcriptome analysis

Bioinformatics analysis of the datasets was as previously described and is publically available [16]. Analysis of OPMD-deregulated ECM pathways was performed with DAVID, functional annotation clustering tool [17], and statistical analysis of deregulated pathways was performed with *Globaltest*[18]. PCOLCE expression values were obtained from a list of OPMD-deregulated top tables (*p* < 0.05), comparing expression levels in *VL* muscles of OPMD and an age-matched control group [16].

For validation studies reverse transcriptase quantitative-PCR (RT-qPCR) was performed on mRNA from either *TA* muscles of OPMD model and wild type control mice (A17.1 and FVB mice respectively) or murine OPMD model myotube cultures (wild type IM2 and mutant Ala17). RNA extraction, cDNA synthesis and RT-qPCR analysis were performed as previously described [16]. Primers used in RT-qPCR are listed in Table 2. Statistical analyses of fold-changes were performed in Excel. Primers used for individual genes were designed using the Primer3 search engine at http://www-genome.wi.mit.edu/cgi-bin/primer/primer3.cgi/ and are listed in Table 2.

**Table 2:** Primers used for quantitative RT-qPCR

Mouse Pabpn1	Fw CCGGACCACCAACTACAACA
	Rv TTGCCCAGGAGCCTGAAG
Mouse Pcolce	Fw CGCTCTCGATTCTACAGTGGTTT
	Rv AGGAGAGAGAGGAGGATTATGTGTGAA
Mouse Desmin	Fw CGCTCTGACCCTAAGACAGG
	Rv GGAAGCCTCTGGTGTCTCTG
Mouse Hprt	Fw TCCAAGCCGGACCTCACA
	Rv CTGAGTCAAGTCTGAAACCTTGGA
FLAG-hPABPN1	Fw CGTCGTGATTAGCGATGATG
	Rv TTTTCCAAATCCTCGGCATA

## Results

### Significant ECM deregulation in OPMD

**Figure 1:**
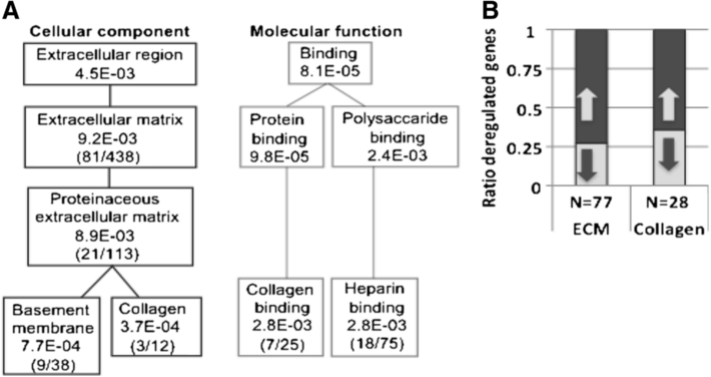
**ECM down-regulation in OPMD patients. A.** Gene ontology trees of the extracellular region component and binding molecular function of ECM localized components are significantly deregulated in the human OPMD transcriptome. P-values indicated for each GO-term were calculated by Global test. Between hyphens the numbers of deregulated genes (left) and the total number of annotated genes per GO-term (right) are indicated. **B.** ECM and Collagen encoding genes are predominantly deregulated in OPMD. Histogram shows the percentage of up-regulated and down-regulated genes in the ECM and Collagen GO categories.

In a recent cross-species study for OPMD we demonstrated that similar molecular systems are deregulated in animal models that overexpress expPABPN1 and in OPMD patients [16]. In addition, similar genome-wide transcriptional changes were also found between *VL* muscles from OPMD patients and a murine muscle cell model system where expPABPN1 was expressed in skeletal myotube cultures at levels similar to endogenous Pabpn1 [14]. The ECM pathway in *Kyoto Encyclopedia of Genes and Genomes* (KEGG) was consistently deregulated in OPMD patients and model systems [14,16]. In KEGG the ECM functional group is only partly annotated compared with Gene Ontology (GO) therefore, we performed a *Globaltest* (GT) analysis of ECM-associated GO terms. In the GO Cellular Component, the *basement membrane* and *Collagen* functional groups were found to be significantly deregulated in OPMD (Figure 1A, left panel). In the Molecular Function GO, the ECM*-associated Collagen binding* and *Heparin binding* functional groups were also significantly deregulated in OPMD (Figure 1A, right panel). In the *ECM* and in the *Collagen* functional groups the majority of the OPMD-dysregulated genes were up-regulated (72% and 64%, respectively; Figure 1B). This observation suggests that muscle fibrosis in OPMD is associated with ECM dysregulation.

**Figure 2:**
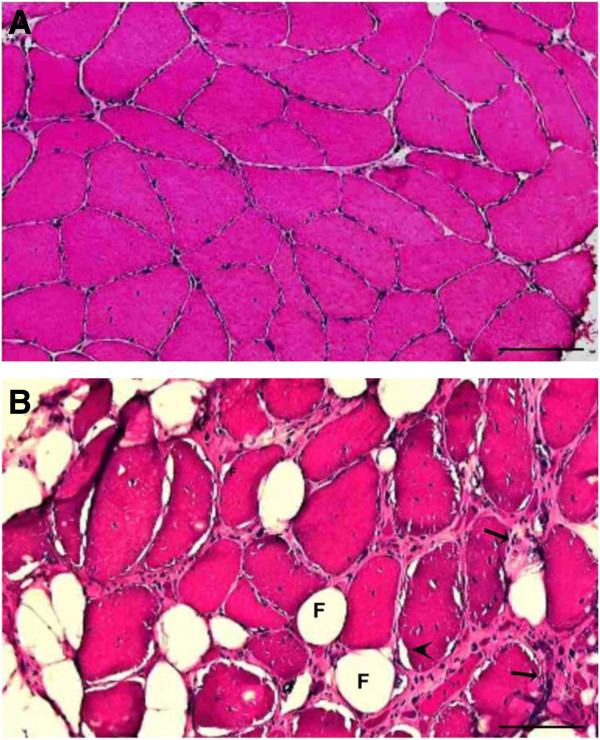
**Transverse cryosections of *VL *muscle biopsies from an OPMD patient over a 6 year interval. A)** Biopsy taken at the time of first presentation in the clinic. **B)** Biopsy of the same muscle as in **(A)** taken 6 years later. Sections were stained with haematoxylin-eosin. Muscle fibrosis is evidenced by fat infiltration (F), vacuole with basophilic rim (arrowhead) and thickening of the ECM (arrows). Scale bar equals 100 μm.

In our OPMD cohort, limb-girdle muscles were severely affected [19]. *Vastus lateralis* muscles from our patients showed progressive muscle degeneration (Figure 2). Longitudinal sampling from the same patients reveals that initially muscle histology was not severely affected, but after 6 years fat infiltration, rimmed vacuoles and ECM thickening was found (Figure 2B). Taken together, this muscle pathology indicates fibrosis. Therefore, in this study we focused on the contribution of ECM dysregulation in muscle fibrosis in OPMD.

### Pcolce expression is consistently down-regulated in a murine myotube culture model of OPMD

**Figure 3:**
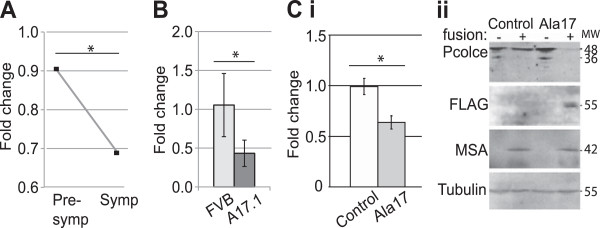
**PCOLCE expression levels are down-regulated in OPMD patients and in a muscle cell model system. A**. Plot shows expression levels of *PCOLCE* in biopsies from pre-symptomatic muscle that carry expPABPN1 but show no disease symptoms (N = 4) and OPMD symptomatic samples (N = 5). Fold change was calculated by reference to age-matched controls (N = 19). Only in the symptomatic group PCOLCE is significantly deregulated (*p < 0.05). **B**. RT-qPCR analysis of Pcolce mRNA levels in *TA* muscles of wild-type (FVB) and A17.1 OPMD model mice at 26-weeks of age. Fold change was calculated after normalisation to the *Hprt* housekeeping gene. Average scores are from 6 mice. Asterisk indicates significant decline (*p < 0.05). **C**. Pcolce levels in myotube cultures expressing expPABPN1-FLAG (Ala17) at a level similar to endogenous Pabpn1. (i) RT-qPCR analysis of Pcolce mRNA expression in myotube cultures of IM2 parental and Ala17 transgene containing cells. Fold change was calculated after normalisation to the *Hprt* housekeeping gene mRNA. Variations between fusion efficiencies were eliminated after normalisation to expression levels from the muscle specific actin (*Acta1*) gene. Averages are from 3 biological replicates. Asterisk indicates significant decline (*p < 0.05). (ii) Western blot analysis of soluble protein extracts from unfused (-) and 4-day fused (+) IM2 and Ala17 cultures. The blot was incubated with anti-Pcolce antibody. Human PABPN1 is detected with an anti-FLAG antibody, the ACTA1 (MSA) protein is a marker for myotube differentiation and tubulin is used as a loading control.

Among the dysregulated ECM components, we found a significant decrease in the expression of procollagen C-proteinase enhancer 1 (*PCOLCE*) in muscle biopsies from OPMD patients compared with an age-matched control group (Figure 3A). The expression of *PCOLCE* in OPMD correlated with muscle symptoms and decreased between carriers of expPABPN1 at a pre-symptomatic stage and at a symptomatic stage (Figure 3A). In order to extend these observations, *Pcolce* levels were determined in a mouse model of OPMD that overexpresses expPABPN1 [10]. As in the case of OPMD patients, *Pcolce* mRNA expression in the OPMD mouse model system was significantly reduced (Figure 3B). In addition, we also found reduced *Pcolce* expression in a murine cellular model for OPMD (Figure 3C). In this cell culture model system, expPABPN1 is fused to a FLAG epitope and is stably expressed in fused immortalized skeletal muscle myotube cultures at levels similar to endogenous *Pabpn1*[14]. Also, in this cell model we verified a decrease in Pcolce protein accumulation (Figure 3Cii). Full length Pcolce (48 kDa) is post-transnationally cleaved generating a 36 kDa polypeptide, corresponding to a secreted form of this protein [20]. Extracellular Pcolce enhances processing of fibrilar procollagen [12,20,21]. Both the full length and the cleaved polypeptides were detected in the control muscle cell culture, but the cleaved polypeptide was differentially expressed between myoblasts and fused myotube cultures (Figure 3Cii). In cultures expressing the Ala17 expPABPN1 transgene the accumulation of soluble full-length Pcolce protein decreased and the cleaved polypeptide was below the level of detection (Figure 3Bii). This suggests that expPABPN1affects PCOLCE protein accumulation in muscle cells.

### Nuclear Pcolce co-localises in nuclear aggregates and co-immunoprecipitates with expPABPN1

**Figure 4:**
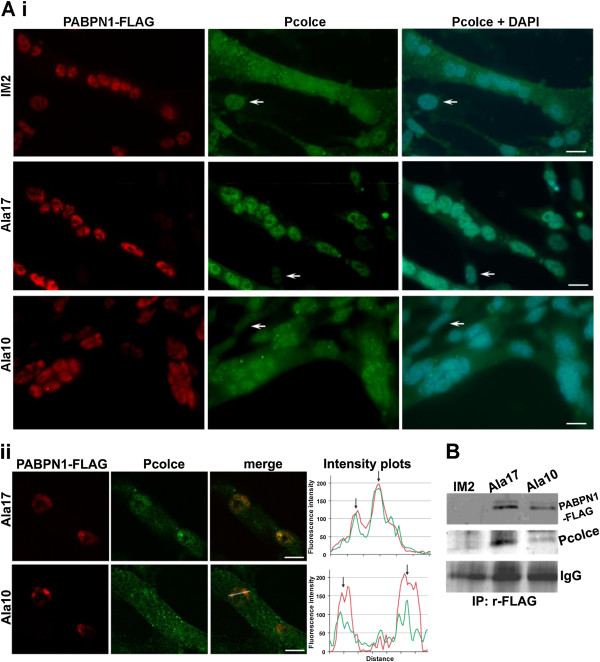
**Co-localisation and co-immunoprecipitation of nuclear Pcolce with expPABPN1 in myotube cultures. A**. Pcolce sub-cellular localisation in myotube cultures. (i) Parental IM2, and those stably expressing Ala17- or Ala10- FLAG-tagged PABPN1were co-stained for Pcolce and PABPN1. PABPN1 was detected with 3 F5-VHH llama antibody and anti-mouse Alexa 594. Rabbit-anti-Pcolce antibody was detected with Alexa 488. Nuclei are counterstained with DAPI. Scale bars equal 20 μm. Arrows indicate unfused myoblast cells. (ii) Single confocal microscope section of myonuclei in Ala17 or Ala10 cell clones. Co-localisation of the two proteins is shown in the merged image. Plots of fluorescence intensity distribution (measured across the line in the merged image) show co-localisation of Pcolce and PABPN1 in fluorescent foci (arrows). Representative myonuclei with PABPN1 foci are shown. Scale bars equal 5 μm. **B**. Immunoprecipitation of PABPN1 in protein extracts from parental IM2, Ala17- and Ala10- PABPN1-FLAG myotube cultures was performed with rabbit-anti-FLAG antibodies. Immunoblotting was conducted with mouse-anti FLAG antibody or rabbit anti-Pcolce. IgG HC is used as a loading control.

The presence and location of Pcolce was then determined in muscle cell cultures using immunolabelling. In parental un-fused skeletal myoblast cells (IM2) Pcolce was predominantly present within the nucleus, whereas in fused myotube cultures a substantial amount was also detected within the cytoplasm (Figure 4A). This suggests that Pcolce sub-cellular localisation changes between upon myoblast differentiation to fused myotubes. In contrast with control cultures, myotubes expressing human expPABPN1 (Ala17), Pcolce was predominantly located within the nucleus (Figure 4A). Pcolce sub-cellular localisation in myotube cultures that stably express wild type PABPN1 (Ala10) was similar to that in parental IM2 cultures (Figure 4Ai). Taken together, these data indicate that overexpression of mutant expPABPN1 but not PABPN1 alters PCOLCE sub-cellular localisation. Furthermore, these observations suggest that expPABPN1 causes accumulation of nuclear PCOLCE.

Formation of expPABPN1 nuclear aggregates is the hallmark of OPMD. Therefore, we next investigated whether PCOLCE co-localises with PABPN1 within nuclear aggregates. Aggregated PABPN1 forms distinct fluorescent foci and co-localization with candidate proteins could be determined using intensity distribution plots [22]. We identified that Pcolce co-localises with PABPN1 in fluorescent foci in the skeletal myoblast cell model system (Figure 4Aii). Co-localization was more pronounced in fluorescent foci formed by expPABPN1 compared with those consisting of wild type PABPN1 (Figure 4Aii). This suggests that PCOLCE binds to nuclear PABPN1 and with a preference for expPABPN1 compared to the wild type protein. Analysis by immunoprecipitation of soluble protein extracts from myotube cultures with an anti-FLAG antibody that recognises transgene-derived Ala10- or Ala17- PABPN1-FLAG, revealed that Pcolce co-immunoprecipites with PABPN1 (Figure 4B). Full-length (48 kDa) Pcolce protein co-immunoprecipitated with both Ala10- and Ala17- PABPN1-FLAG, but there was a clear enrichment in binding between Pcolce and mutant Ala17-PABPN1 compared to that with wild type Ala10 PABPN1 (Figure 4B). These results show that Pcolce binds to soluble PABPN1 but with a higher affinity to expPABPN1.

### Pcolce localisation is reduced at the ECM and is retained in the nucleus in the OPMD mouse model and in OPMD *VL* muscles

**Figure 5:**
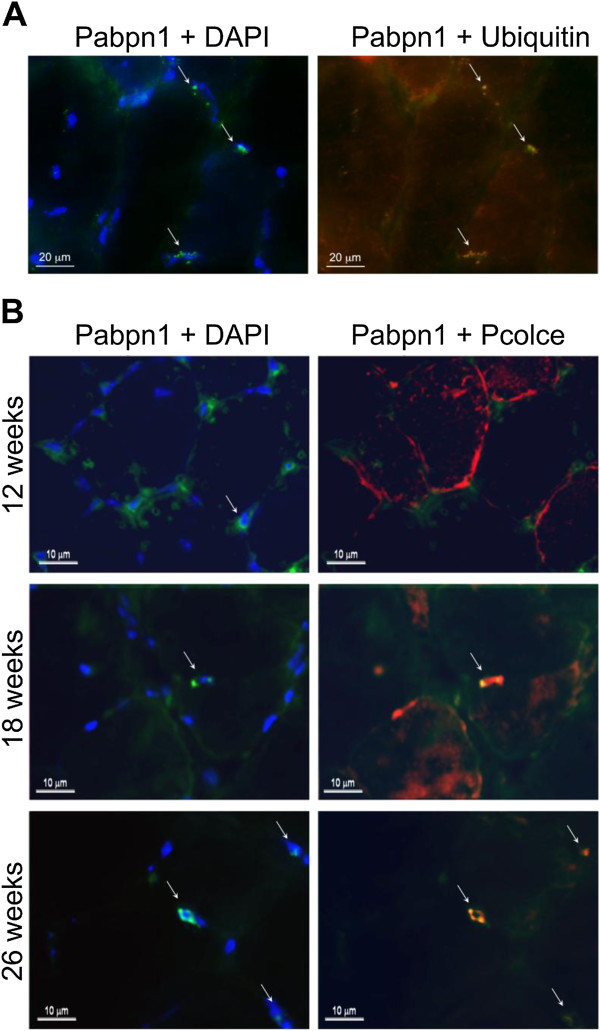
**Pcolce protein is sequestered within nuclear aggregates of the mouse OPMD model system.** Immunofluorescence staining of *TA* muscle sections derived from A17.1 OPMD model mice. **(A)** PABPN1 aggregates were recognised by the high fluorescence intensity of nuclear PABPN1 and their co-localisation with ubiquitin (arrows). Scale bars are 20 μm. **(B)** Co-localisation of Pcolce with PABPN1 was conducted on *TA* muscle sections from 12, 18 and 26 week old A17.1 mice. PABPN1 is visualised with 3 F5-VHH and mouse anti-myc antibodies (green), Pcolce or ubiquitin are visualised with rabbit antibodies and are marked in red. Co-localisation between Pabpn1 and Pcolce is shown in yellow in the merged image. The nucleus is counterstained with DAPI (blue). Nuclear aggregates are indicated with arrows. Scale bars equal 10 μm. Immunostaining was performed on sections from 3 mice. Shown are representative images.

In order to demonstrate a pathological relevance of the change in PCOLCE sub-cellular localisation and nuclear enrichment observed in the skeletal myoblast model system of OPMD (Figure 4), we next investigated PCOLCE localization in cryosections of the *Tibialis anterior* (*TA*) muscle from the A17.1 OPMD mouse model, and in *VL* muscles from an OPMD patient. In the mouse model, the human Ala17 expPABPN1 is overexpressed in skeletal muscles with muscle weakness detected in 5–6 month old animals [10,11]. Aggregated expPABPN1 fluorescent foci co-localised with ubiquitin (Figure 5A). Pcolce co-localisation with expPABPN1 aggregate fluorescent foci was found in muscles from 18 and 26-weeks but not in 12-week old A17.1 mice (Figure 5B). This observation supports our findings from *in vitro* analyses, and suggests that Pcolce is sequestered within nuclear PABPN1 aggregates after these structures have formed.

**Figure 6:**
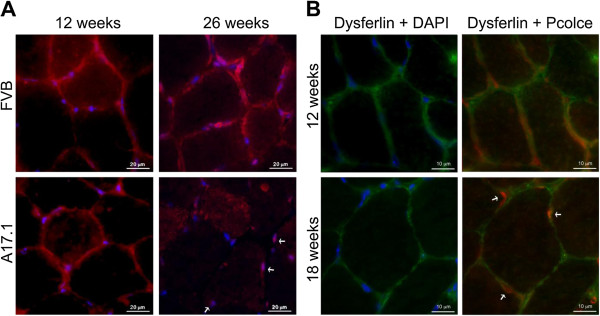
**Reduction of Pcolce signal at the ECM in the OPMD model mice. (A)** Immunofluorescence staining of *TA* muscle sections of 12- and 26-week old wild type (FVB) and A17.1 OPMD model mice was conducted with anti-Pcolce antibodies (red). DAPI staining of nuclear DNA (blue) is merged. Some nuclei with entrapped Pcolce are indicated with arrows. Scale bars are 20 μm. **(B)** Immunolocalisation of Pcolce in sections from 12 and 18-week old A17.1 mice was conducted in combination with anti-dysferlin (green), which marks the sarcolemma of skeletal muscles. Some nuclei with entrapped Pcolce are indicated with arrows. Scale bars are 10 μm. Immunostaining was performed on sections from 3 mice for each genotype. Shown are representative images.

**Figure 7:**
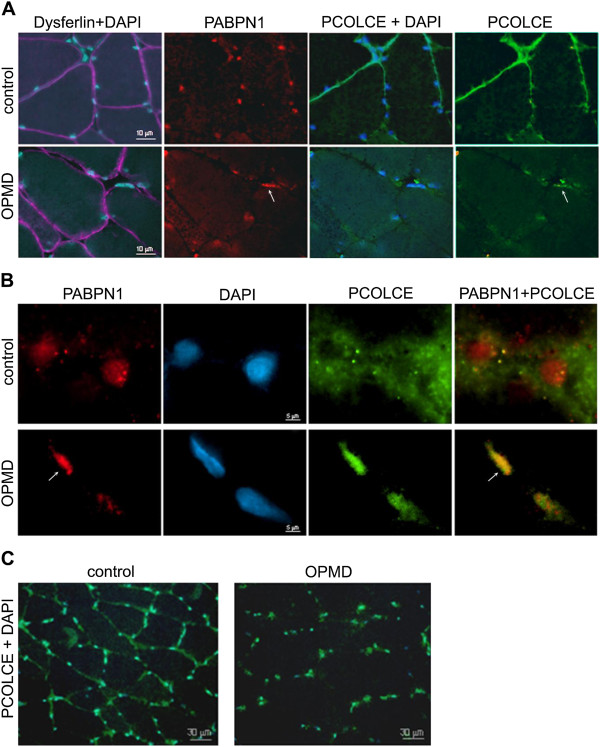
**PCOLCE sub-cellular localisation in OPMD and control muscle biopsies.** Muscle cryosections were co-stained for PABPN1 (red; 3 F5-VHH) and PCOLCE (green; 3 F5-VHH). Nuclei were counterstained with DAPI (blue). Arrows point to nuclear PCOLCE that colocalizes with aggregated PABPN1. (**A**) The sarcolemma is highlighted by staining for dysferilin (purple). Scale bar equals 10 μm. **(B)** Immunofluorescence after KCl treatment. Treatment with 1 M KCl disturbs localisation of soluble proteins whilst aggregates remain intact (arrow). Co-localisation between PABPN1 and PCOLCE is shown in the merged image. Scale bar equals 5 μm. **(C)** Changes in PCOLCE sub-cellular localisation shown at low magnification. PABPN1 and DAPI mark nuclei. Scale bar equals 30 μm.

In addition to Pcolce co-localisation in PABPN1 nuclear aggregates, we observed a concomitant decrease in the levels of this protein at the ECM in mice aged from 12 to 26 weeks (Figure 6A). In *TA* sections from wild type (FVB) mice, Pcolce signal at the ECM did not change between 12 and 26 weeks (Figure 6A), while in muscles from A17.1 OPMD model animals a decrease in extracellular Pcolce signal was evident from week 18 of age (Figure 6B). In contrast to the decrease in extracellular Pcolce signal, the amount of nuclear Pcolce remained high in 18 and 26 weeks old A17.1 muscles (Figure 6B). This confirms that expression of expPABPN1 induces a change in PCOLCE sub-cellular localization in muscle. Importantly, the change in Pcolce sub-cellular localization in the A17.1 OPMD mouse correlated with initiation of muscle weakness in this model system. This suggests that POLCE sub- cellular distribution is associated with muscle weakness. In addition, the extracellular PCOLCE signal in OPMD *VL* muscles was reduced compared with its localization in a healthy control (Figure 7A). Furthermore, in OPMD but not in the healthy control nuclear PCOLCE showed intense staining (Figure 7A) whereas the control Dysferlin signal showed no severe abnormalities in these fibres from the OPMD patients (Figure 7A).

To confirm that nuclear PCOLCE co-localizes with aggregated PABPN1, muscle sections were treated with 1 M KCl to remove most soluble nuclear proteins. Aggregated nuclear proteins are resistant to KCl treatment and are present as intense fluorescence foci, whilst soluble proteins are observed as a diffuse signal. However, it should be noted that the severity of the treatment with 1 M KCl disrupts the morphology of the cell nucleus. Nevertheless, in the section of OPMD patient muscle (Figure 7B, lower panel), two nuclei are shown. In one nucleus the PABPN1 inclusion encompasses most of the nucleus within which PCOLCE is co-localized with aggregated PABPN1 (Figure 7B, lower panel, arrow). In the other nucleus, accumulation of aggregated PABPN1 is far more limited and PCOLCE co-localization is also reduced. This suggests that PCOLCE entrapment within PABPN1 nuclear aggregates is consistent between OPMD patient and cellular (Figure 4A) and animal (Figure 5) model systems. In marked contrast to the paucity of nuclear aggregates in OPMD myonuclei (1-5%; [8,23]), the reduction in extracellular PCOLCE and its nuclear enrichment was uniformly distributed throughout the muscle section from the OPMD patient (Figure 7C). Collectively, our data from the OPMD cell and animal model systems combined with that obtained from patient *VL* muscle biopsy samples, highlights a change in the sub-cellular distribution of PCOLCE in OPMD.

**Figure 8:**
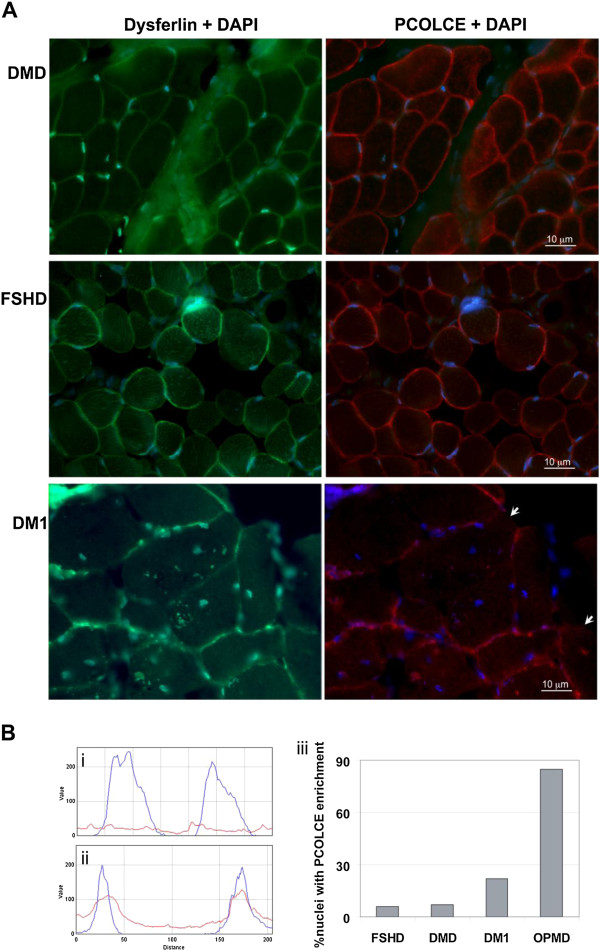
**Nuclear localisation of PCOLCE is enriched in OPMD compared to other muscular dystrophies. A**. Sub-cellular localisation of PCOLCE in DMD, FSHD and DM1 patient muscle. Cryosections were stained for PCOLCE and Dysferlin. Dysferlin shows a sarcolemmal localisation. In DMD and FSHD biopsies there is no clear reduction of PCOLCE at the ECM. In DM1 areas with reduced PCOLCE at the ECM are indicate with arrows. Scale bar equals 10 μm. **B**. Accumulation of PCOLCE in myonuclei was quantified using overlap in intensity distribution between DAPI (blue) and PCOLCE (red). Plots show two nuclei without enrichment with nuclear PCOLCE (i) or with PCOLCE enrichment (ii). Histogram (iii) shows the quantification of 100 nuclei from FSHD, DMD, DM1 and OPMD muscle biopsies.

In order to determine whether change in PCOLCE sub-cellular distribution is a general phenomenon of muscle wasting, PCOLCE sub-cellular localisation was studied in *VL* muscles of patients with DMD, myotonic dystrophy type 1 (DM1) and Facioscapulohumeral muscular dystrophy (FSHD). Histological analysis of muscle biopsies from all these conditions revealed widespread fibrosis and ECM thickening throughout muscle fibres (Figure 8A). In this same series of sections, PCOLCE staining was uniformly localized to the ECM in the DMD and FSHD samples (Figure 8A). A reduction in PCOLCE at the ECM and enrichment of this protein within the nucleus was observed in DM1 (Figure 8A). A quantitative comparison of nuclear PCOLCE across these 4 muscle disorders demonstrated PCOLCE nuclear enrichment in OPMD and DM1 compared to DMD and FSHD (Figure 8B). This suggests that nuclear entrapment of PCOLCE is not a general phenomenon in muscle degeneration.

## Discussion and conclusion

ECM defects are associated with muscle fibrosis of muscular disorders, and are usually detected at a progressed state of the disease. Muscular dystrophies including OPMD are characterised by the presence of fibrotic muscles. Deregulation of the ECM is found in inherited degenerative muscle diseases such as DMD, FSHD and α-sarcoglycan deficiency [24-26]. ECM thickening in DMD patients are associated with significant dysregulation of collagen and other ECM protein genes [25]. Muscles from symptomatic patients show ECM thickening that is typical of fibrotic tissue. Collagen type I and III are major components of the ECM in skeletal muscle [27], and in inherited muscular dystrophies an up-regulation of collagen-encoding genes is associated with thickening of the ECM. ECM thickening affects muscle contraction and extracellular signal transduction and increased inflammation [5]. In DMD patients, ECM thickening is associated with up-regulation of collagen encoding genes [25]. Similar to other muscle disorders, in OPMD we found significant molecular dysregulation of ECM-associated genes. Since dysregulation of ECM encoding genes in different cell types are often associated with ECM thickening and profibrotic situations [28], it is highly likely that the transcriptional changes in the ECM functional group in OPMD are associated with muscle degeneration.

We identified consistent down-regulation of PCOLCE in OPMD patient and cellular and mouse model systems of this condition. In carriers of expPABPN1, down-regulation was found in symptomatic patients but not at the pre-symptomatic stage of the disease. This suggests that the change in PCOLCE expression in muscles is associated with muscle degeneration. PCOLCE is down-regulated in OPMD and is found in PABPN1 nuclear aggregates. Products of dysregulated genes in model systems expressing expPABPN1 often co-localize with PABPN1 within nuclear aggregates [16,29]. The mechanism as to how this occurs is not fully understood. However, it is possible that a feedback-forward regulatory mechanism between protein aggregation and mRNA accumulation is disturbed when aggregates are formed.

The procollagen I peptide is proteolytically processed to form collagen fibres. Extracellular PCOLCE stimulates the function of procollagen C-proteinase, thereby leading to increased cleavage and processing of procollagen I proteins [21]. Pcolce deficient mice exhibit ECM thickening, aberrant procollagen peptide processing and deformed collagen fibril morphology [30]. Taken together these processes suggest that a reduction in PCOLCE at the extracellular matrix would diminish collagen fibrillogenesis and hence negatively affect ECM maintenance [30]. Of relevance to muscle disorders, Pcolce down-regulation has been found in Myostatin-knockdown mice, and was associated with reduced collagen content and muscle fibrosis [31]. It is therefore likely that in OPMD a reduced expression of PCOLCE causes ECM thickening and consequently contributes to muscle fibrosis.

PCOLCE is a secreted protein. It is synthesized in the lumen of the ER and is transported to the extracellular compartment via the Golgi apparatus and associated secretion pathway [12]. In addition to its known extracellular location at the ECM, we found PCOLCE to also be present within the cell nucleus. We found that PCOLCE sub-cellular localization is dynamic in muscle cells, and changed upon myoblast differentiation to fused myotubes. In degenerated OPMD muscles, a reduction in extracellular PCOLCE is associated with retention of this protein within the nucleus. Since PCOLCE protein biosynthesis is at the secretary Golgi apparatus, this suggests that PCOLCE may shuttle from the extracellular into the cell nuclear compartment. However, the mechanism regulating PCOLCE sub-cellular localization remains to be investigated.

We found that PABPN1 co-immunoprecipitates with PCOLCE (Figure 4B). Since PABPN1 is predominantly localized within the cell nucleus and we found co-localization of PABPN1 and PCOLCE within this sub-cellular compartment (Figures 4A, 5 and 7B), we conclude that PCOLCE binds to nuclear PABPN1 and consequently is entrapped in PABPN1 aggregates.

Co-localization between PCOLCE and aggregated PABPN1 was found to be consistent between cellular and animal model systems of OPMD as well as within OPMD patient muscle samples. Protein entrapment within PABPN1 inclusions is a gradual process where proteins such as ubiquitin or poly(A) polymerase co-localize with small and intermediate sized aggregates, whilst others like heat-shock protein 70 (HSP70) are recruited to large and non-reversible aggregates [22]. Since only limited co-localization of PCOLCE and PABPN1 was found in OPMD nuclei with reduced amounts of aggregated PABPN1 (Figure 7B), it is likely that PCOLCE, as in the case of HSP70, is also recruited to inclusions but not to the intermediate sized PABPN1 aggregated structures.

PABPN1 is a regulator of mRNA processing [32-35]. A functional role for nuclear PCOLCE is, as yet, unknown. PCOLCE contains RNA binding motifs and a role in RNA stabilisation has been suggested [36]. Here we show that PCOLCE binding to PABPN1 is enriched in cells expressing mutant expPABPN1. This suggests that PCOLCE binding to PABPN1 is of relevance to OPMD pathogenesis. It is possible that due to the alanine expansion in expPABPN1 the dynamics of the PCOLCE-PABPN1 complex is reduced and that this in turn could negatively affect PABPN1 function.

In contrast to the paucity of PABPN1 nuclear aggregate formation in affected myonuclei [8,23], we found a consistent reduction in ECM-localised PCOLCE levels across muscle fibres of OPMD patients, and in a mouse model of this disease. This consistency suggests that this feature could be employed as a marker of affected muscles in OPMD patients. In a skeletal myoblast cell model system expressing expPABPN1, we observed accumulation of nuclear PCOLCE and co-localisation of PCOLCE with aggregated PABPN1. This observation was confirmed in OPMD patient muscle biopsy samples, where nuclear PCOLCE accumulation was more prominent compared with that seen in a healthy control or other muscular dystrophies, namely DMD and FSHD. As both OPMD and MD1 are classified as late onset disease conditions whilst DMD starts within childhood and symptoms in FSHD are typically observed from around 20 years of age, it is possible that the change in PCOLCE sub-cellular localisation may be of greater relevance to late onset muscle disorders. However, the contribution of dysregulation of PCOLCE sub-cellular localisation to disease pathophysiology remains to be demonstrated.

## Competing interests

The authors declare that they have no competing interests.

## Authors’ contributions

Microarray and RNA analysis was conducted by ES, AV. Protein assays and immunofluorescence analysis were done by VR and ES. Patient muscle biopsy samples were provided by BvdS and BvdE. Mouse tissue samples were provided by CT and GD. The myoblast OPMD model system was generated and provided by SR and MNA. Project planning and manuscript preparation was undertaken by VR, MA and SvdM. All authors read and approved the final manuscript.

## Pre-publication history

The pre-publication history for this paper can be accessed here:

http://www.biomedcentral.com/1471-2377/13/70/prepub
